# Molecular Monitoring in Soil Bioremediation: From Genetic Potential to Verified Pathway Operation

**DOI:** 10.3390/ijms27073111

**Published:** 2026-03-29

**Authors:** Mariusz Cycoń

**Affiliations:** Department of Microbiology, Faculty of Pharmaceutical Sciences, Medical University of Silesia, Jagiellońska 4, 41-200 Sosnowiec, Poland; mcycon@sum.edu.pl

**Keywords:** soil bioremediation, biodegradation pathways, molecular evidence framework, soil microbiome, gene expression, multi-omics integration, pathway verification, stable isotope probing

## Abstract

Sequence-based tools have greatly improved the molecular description of soil bioremediation, but detection alone cannot confirm that a contaminant is being degraded by a defined pathway. In soils, bioavailability limitations, redox microsites, relic DNA, gene mobility, and community restructuring can decouple gene presence from reaction flux. This review synthesizes an operational framework that separates three inferential levels: pathway potential, in situ activity, and verified pathway operation. The framework links inoculant fate, functional gene abundance, gene expression, pathway reconstruction, stable isotope probing, and targeted chemical analysis under explicit quality assurance, quality control, and decision rules. Particular attention is given to distinguishing parent compound loss from mineralization and detoxification and to using isotopic attribution when functional redundancy or inoculant-native overlap obscures agency. Instead of being presented as conceptually new, these principles are organized into a practical workflow for soil systems. This structure clarifies what can be discerned from genes, transcripts, proteins, metabolites, and transformation products at each evidentiary tier and provides a conservative basis for integrating multi-omics with mechanistic and quantitative interpretation.

## 1. Introduction

Soil bioremediation is one of the few remediation strategies that can be implemented in situ at scale without transferring contaminated material to expensive ex situ treatment systems. Its effectiveness is governed less by nominal microbial catabolic capacity than by the physical and chemical constraints imposed by the soil matrix. Diffusion limitation, sorption to mineral and organic phases, microscale redox stratification, and uneven access to electron acceptors determine which contaminant fraction is actually available to microorganisms at a given time [1,2]. As a result, before-and-after concentration comparisons are easy to overinterpret. Parent-compound decline may reflect sequestration, volatilization, dilution, or partial biotransformation rather than mineralization, and similar concentration profiles can arise from fundamentally different processes [1,3]. Recent reviews of research on agrochemical-contaminated soils and enzyme-mediated bioremediation have reinforced the notion that successful remediation depends not only on the presence of degraders but also on the relationship between degradative enzymes, their encoding genes, microbial hosts, and environmental constraints that determine whether those enzymes operate in situ [4,5]. This broader understanding strengthens the need for a framework that separates enzymatic capacity from verified pathway operation in soil systems. 

**Figure 1:**
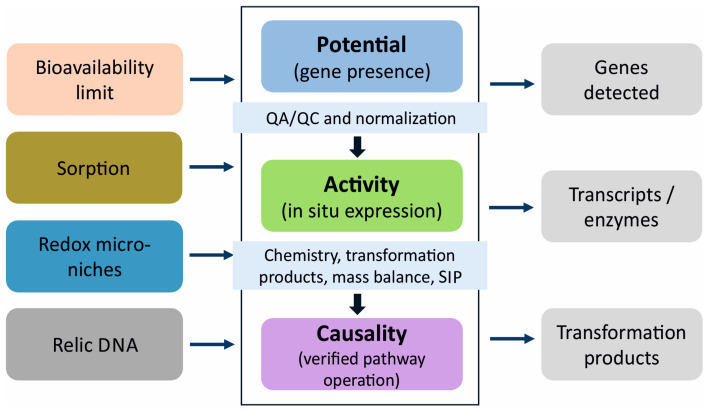
Soil constraints and evidence tiers in molecular monitoring. Conceptual summary of the soil processes that decouple pathway possession from realized transformation and of the stepwise progression from potential, to activity, to causal pathway confirmation. SIP: stable isotope probing; QA: quality assurance; QC: quality control.

Molecular measurements are, therefore, most informative when interpreted together with contaminant fate and site geochemistry rather than as stand-alone inventories of taxa or genes [2,6,7,8]. The rapid expansion of quantitative PCR (qPCR), droplet digital PCR (ddPCR), metagenomics, metatranscriptomics, proteomics, metabolomics, and isotope-based approaches has greatly improved detectability, but detectability does not equate to functional effects. The soil constraints that decouple molecular signals from realized biodegradation, together with the gated progression from potential to activity and causality, are summarized in Figure 1. In soils, pathway possession is often decoupled from pathway deployment by bioavailability, redox conditions, physiological stress, and spatial structure [2,3]. Nonliving nucleic acids intensify this problem because a substantial fraction of extracted DNA may derive from inactive or extracellular pools, obscuring the temporal patterns relevant to remediation assessment [9,10,11,12,13,14]. Approaches that enrich viable fractions can improve inference, but they remain matrix-dependent and do not confirm in situ activity [9,10,15]. Recent reviews of agrochemical remediation and microbial enzyme systems further reinforce the notion that molecular interpretation must consider mechanisms, environmental context, and the chemistry of transformation [4,5,16].

The conceptual contribution of this review is not a general claim that DNA-based evidence alone is insufficient, a point that is already well-established in the literature. Rather, this study offers a three-level evidence framework that separates pathway potential, in situ activity, and verified pathway operation and links progression between these levels to explicit quality assurance (QA)/quality control (QC), normalization, pathway-consistency, and attribution rules. Instead of a descriptive survey of methods, this review offers a decision framework for interpreting molecular data in heterogeneous soil systems. Accordingly, it is organized around four guiding questions: What minimum evidence supports pathway potential in soil? What evidence shows that the pathway is engaged under in situ conditions? What evidence is required before attributing observed chemical change to a defined pathway? How should molecular signals be integrated with transformation products, isotopic attribution, and site heterogeneity to support causal inference? In this review, potential, activity, and causality are treated as noninterchangeable inferential categories. The objective was to formalize a conservative framework for linked molecular monitoring in which inoculant fate, functional gene abundance, gene expression, pathway reconstruction, and chemical confirmation are interpreted only at the evidentiary level that the available data can support. Throughout this review, four related terms are used in a strict sense: Biodegradation refers to the microbe-mediated transformation of a contaminant, whether partial or complete. Biotransformation refers specifically to the structural conversion of a parent compound without implying pathway completion, mineralization, or detoxification. Mineralization is the conversion of contaminant-derived atoms to inorganic end products such as CO2, CH4, NH4+, or other terminal mineral forms, depending on the element and redox context. Detoxification denotes a reduction in biological hazard and is not inferred from parent-compound loss alone.

## 2. Contaminants and the Soil Biodegradation Context

The central issue in bioremediation monitoring is not contaminant classification per se but the extent to which soil conditions permit pathway induction and pathway completion. The interpretive value of metagenomics and other omics layers, therefore, depends on whether they are anchored to contaminant accessibility, local redox structure, and transformation chemistry [6,7,8,9,10,11,12,13,14,15,16,17,18,19,20,21,22,23,24,25].

For petroleum hydrocarbons, bioavailability is usually the primary bottleneck, especially in aged soils with strong hydrocarbon sorption [26,27,28]. In refinery soils, mineralization of labeled hydrocarbons by autochthonous microbiota can be measurable and can increase after stimulatory amendments [29]. Even so, canonical markers such as *alkB*, which encodes alkane 1-monooxygenase, do not predict reaction rate or pathway completion alone. In some degraders, initial alkane oxidation is instead mediated by CYP153, a family of soluble cytochrome P450 alkane hydroxylases involved in the oxidation of short- to medium-chain n-alkanes that expands the range of pathway initiators that must be considered [30]. These cases illustrate why marker detection must be interpreted in the context of contaminant accessibility and site geochemistry rather than treated as direct evidence of ongoing degradation [31,32,33,34,35,36,37,38,39,40].

For polycyclic aromatic hydrocarbons (PAHs), the analytical burden is higher because both pathway initiation and downstream ring-cleavage steps must be considered. Hydrophobicity, sorption, and diffusion barriers reduce microbial access, whereas prolonged residence time shifts PAHs into sequestered fractions [41,42,43]. Marker panels centered on a single initiation gene therefore risk oversimplifying pathway status [44,45,46]. The commonly used *pah-rhd* markers target the alpha subunit of PAH ring-hydroxylating dioxygenases, whereas *pahE* (encoding PAHs hydratase-aldolase) has been proposed as an additional functional marker that broadens coverage of aerobic PAH-degrading bacteria [47,48,49]. Metatranscriptomics of phenanthrene-exposed soils has shown that broad community transcriptional responses do not necessarily identify the taxa executing pathway-defining steps, a gap that stable isotope probing (SIP) can resolve by linking substrate-derived carbon to active populations [50,51,52,53,54,55,56].

Among pesticides, atrazine is a model case because pathway genetics and chemical outcomes can be evaluated together [57,58,59]. Bioaugmentation with *Paenarthrobacter* sp. strain AT-5 accelerated atrazine removal while the abundance of *trzN*, which encodes a triazine hydrolase initiating atrazine dechlorination, remained comparatively stable [60]. This pattern illustrates a broader point: gene presence and even stable gene abundance alone cannot explain pathway throughput. Expression data, community interactions, and substrate accessibility remain decisive [61,62,63,64]. Other pesticide systems reinforce this mechanistic diversity. PdmAB is an N-demethylase required for the initial step of *N*,*N*-dimethyl-substituted phenylurea herbicide degradation [65]. However, recent studies of fluorinated pyrethroids and tembotrione show that cytochrome P450-dependent reactions and consortium-level pathway partitioning can shape pesticide biotransformation in soil-derived microbial systems [66,67]. These examples show that degradation in soil may be distributed across multiple enzymatic modules and taxa, even when the parent compound follows a single apparent removal trajectory.

For pharmaceuticals, the central requirement is to distinguish compound disappearance from true mineralization [68,69,70]. For instance, in agricultural soils, diclofenac is considered readily biodegradable [71]; however, labeled sulfamethoxazole studies have demonstrated that rapid removal can occur simultaneously with partial mineralization [72]. Similarly, antibiotic degradation is associated with shifts in microbial community structure and activity [73], as well as dynamic responses in resistance and mobility genes, which are relevant to both interpretation and risk assessment [74,75,76,77,78].

Taken together, these contaminant classes move from diffusion-limited hydrocarbon systems to structurally specific pesticide pathways and to pharmaceutical systems in which parent loss and detoxification are particularly easy to confuse. Across all three contexts, bioavailability can constrain reaction rates even when genetic potential appears substantial, which limits the explanatory power of markers such as *alkB* or *pah-rhd* [79,80]. Redox conditions and coupled biogeochemical processes impose further constraints. In petroleum-contaminated soils, for example, biodegradation can be tightly linked to nitrogen cycling, and adjacent processes may govern reaction rates as strongly as the catabolic pathway itself [81,82]. Molecular signals become quantitatively informative when they are interpreted within biogeochemical or kinetic models that include sorption–desorption, diffusion limitation, electron-acceptor availability, and biomass-specific transformation capacity. In such models, gene abundance constrains pathway capacity, transcript or protein data constrain inducible biological engagement, and contaminant time series constrain realized flux. This integrated approach helps distinguish low transformation rates caused by poor bioavailability from those caused by lack of pathway induction or physiological stress [83,84,85,86,87,88,89].

## 3. An Evidence-Based Framework for Linked Molecular Monitoring

In soil bioremediation, the presence of degraders does not necessarily indicate ongoing degradation. Similarly, a decline in contaminant concentration does not automatically reflect microbial activity. These assumptions can lead to systematic oversimplification. Omics readouts support decision-making only when they strengthen causal inference about contaminant fate and not when they merely enlarge catalogs of taxa and genes [1,3]. A defensible chain of evidence separates three levels: degradative potential, in situ activity, and pathway confirmation that accounts for the observed transformation [62,90].

Gene detection alone is insufficient because pathway presence is often decoupled from deployment in soils, and DNA often records past rather than current function [2]. Relic DNA can constitute roughly 40% of prokaryotic and fungal pools, inflating apparent diversity by up to 55% [11]. Evidence of potential should originate from an explicit pathway hypothesis that spans initiation through completion, supported by validated markers and analytical workflows. Amplicon and metagenomic inferences are sensitive to pipeline choices and thresholds [91,92], and functional surveys can be misleading due to primer bias or incomplete coverage of degradative diversity [47,48]. In situ engagement requires unambiguous transcription, and ideally translation, of pathway modules under exposure conditions. For atrazine, quantitative tracking of *atz* transcripts (involved in atrazine degradation) provides a direct record of pathway induction in soils [61]. Expression distinguishes gene possession from effective use under contaminant and environmental constraints [62], yet transcription alone does not confirm throughput or rates. Rigorous quality assurance (QA) and quality control (QC) are essential, including verification of inhibitor resistance. ddPCR enables absolute quantification but is difficult near detection limits or in variable matrices [93,94,95]. Reverse-transcription ddPCR shows high inhibitor tolerance in soils and plant materials, but workflows still require inhibition testing, recovery controls, biologically meaningful normalization, and transparent reporting [91,95,96]. Correlations between genes and decline are inadequate. Chemical lines of evidence should verify pathway progression through biotransformation products, mass balance, and consistency between metabolite profiles and predicted steps [97,98]. Attributional methods complement chemistry: DNA- or RNA-based SIP links labeled substrate carbon to active microorganisms, while protein SIP assigns incorporation to enzymes, helping disentangle gene possession from gene use when many taxa share potential [90,99,100,101,102]. Viability PCR (vPCR) with propidium monoazide (PMA) can suppress signals from compromised cells and extracellular DNA, but the results are highly sensitive to soil properties and protocol parameters and require cautious interpretation and full reporting [103,104,105,106]. vPCR can support claims about living fractions, but is not a substitute for activity or throughput evidence.

Because universal numerical thresholds are not transferable across soils, contaminants, and analytical platforms, progression between evidentiary tiers should be defined by operational acceptance criteria rather than fixed copy-number cutoffs. To move from potential to activity, a study should show, at minimum, an explicit pathway hypothesis spanning initiation and downstream completion steps, analytically validated marker coverage, acceptable inhibition and recovery controls, and reproducible expression of pathway-critical genes under exposure conditions [11,62,91,95,96]. To move from activity to causal pathway confirmation, the evidentiary bar should rise further: contaminant decline should be accompanied by pathway-consistent transformation products or mass-balance logic, and attribution should be strengthened by DNA, RNA, or protein SIP whenever multiple plausible degraders or competing pathways remain unresolved [90,97,98,102]. Near-threshold signals, poorly constrained normalization, or uncoupling between transcript dynamics and product formation should halt progression to the next tier rather than be interpreted as partial proof. Operationally, the workflow proceeds from a testable pathway hypothesis to quality-controlled marker detection, evidence of in situ pathway engagement, and finally chemical and isotopic verification when causal claims are made. This explicit gating is intended to reduce interpretive drift in both experimental and field bioremediation studies [62,90,91,95,96,102].

## 4. Inoculant Fate in Soil: Persistence, Dispersal, Viability, and Functional Retention

**Figure 2:**
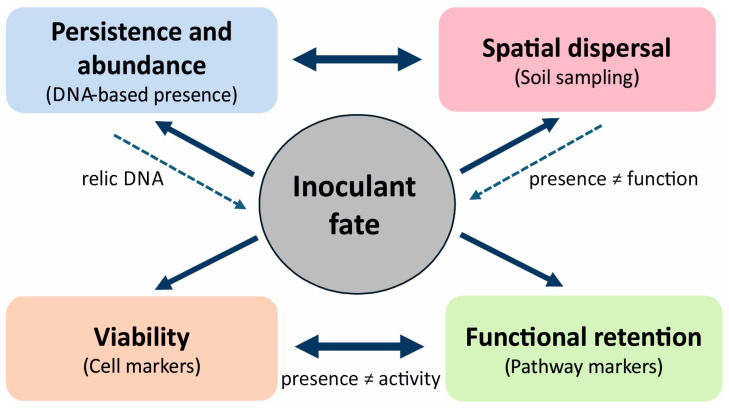
Dimensions of evidence for inoculant fate in the soil. Persistence, dispersal, viability, and functional retention are distinct observables and should not be collapsed into a single abundance-based conclusion.

Bioaugmentation is primarily an ecological intervention and only secondarily a technological treatment. The success of this method depends not on the mere possession of catabolic genes but on the inoculant’s capacity to persist within a heterogeneous soil matrix, access microniches in which contaminants reside, maintain viability and metabolic competence, and retain function under competition, microfaunal pressure, and fluctuating resource availability [1,2]. Empirical studies have consistently reported that inoculant fate is shaped by a combination of priority effects favoring resident taxa, niche overlap, and trophic interactions that cannot be engineered at the genome level alone [3,83,84,89]. Inoculant fate should therefore be decomposed into at least four dimensions for monitoring: persistence and abundance, spatial dispersal, viability, and functional retention, defined as the capacity to execute pathway-critical steps in situ [6,7]. The separation of persistence, dispersal, viability, and functional retention as nonequivalent dimensions of inoculant fate is illustrated in Figure 2.

A recurrent interpretive pitfall is equating DNA detectability with survival. In soils, genetic signals can persist long after cellular integrity is lost, and relic DNA may be sufficiently abundant to mask temporal dynamics and overstate population persistence [11,12,13,14]. Bioaugmentation exacerbates this issue, as inocula are typically introduced at high doses. Rapid loss of viability can therefore leave a strong DNA footprint without functional effects [10,83]. vPCR using PMA reduces the amplification of DNA from cells with compromised membranes and from extracellular pools [10,103,104,106]. However, this technique is highly sensitive to soil properties and protocol parameters, and its outcomes depend strongly on complete and transparent reporting [10,95,96,105]. Even when carefully applied, vPCR can only support inference about the presence of a living fraction and is not evidence of activity or functional retention [9,10,90,102].

An inoculant’s persistence may be transient, yet its introduction can restructure community function. For example, in hydrocarbon-contaminated soil augmented with *Rhodococcus erythropolis* strains CD130 and CD167, community changes were evident within a single day. *Rhodococcus* itself was no longer detected by Day 91, and communities under single-strain treatments converged toward the control. In parallel, the contribution of *Rhodococcus* to *alkH* (encoded alcohol dehydrogenase) abundance declined from 19–77% on Day 1 to 1.0–3.4% by Day 91 and disappeared entirely by Day 182 [107]. This observation has dual implications. First, the disappearance of the inoculant does not preclude lasting ecological effects. Second, the identity of carriers of a functional marker can shift over time; hence, tracking genes without attributing agency may be misleading. Dispersal and microscale colonization are, therefore, not synonymous with functional success.

PAH bioaugmentation studies provide additional evidence for this distinction. In microcosms with contrasting contamination histories, bioaugmentation with *Sphingobium* sp. AM improved phenanthrene removal in freshly contaminated soil but did not enhance the degradation of any of the 10 PAHs in chronically contaminated soil [108]. Despite this observation, pronounced community restructuring occurred, including sustained dominance of Sphingomonadales and shifts in Actinomycetales abundance. These findings assert that establishment and deep community reorganization can occur without a measurable improvement in degradation. Therefore, inoculant fate should be reported independently of chemical endpoints, and community shifts should not be interpreted as evidence of functional retention.

An additional, often underappreciated dimension of inoculant fate is viability under biotic pressure. DNA SIP studies have reported that trophic interactions, including predation, can strongly modulate inoculant persistence and determine which populations assimilate contaminant-derived carbon [1,90,109]. When top-down control eliminates inoculants, monitoring limited to catabolic genes may incorrectly suggest metabolic incompetence. In such cases, inference should be supported by attribution methods that directly demonstrate the assimilation of contaminant carbon [56,90,102,109]. Functional retention distinguishes inoculants that are merely present from those that are causally active; hence, it remains the decisive criterion [3,110]. When metabolism shifts to more accessible substrates or when the expression of catabolic modules declines, function may be lost despite persistence [111,112]. Conversely, when pathway genes are mobile, function may persist within the community even after inoculant loss. In the case of linuron, functional capacity is decoupled from the fate of a single strain as *PromA* plasmids facilitate horizontal transfer of catabolic genes across diverse bacterial genera [100,101,113]. In such systems, monitoring must address both pathway genes and their mobility context, especially under selective pressures that also affect resistance and transfer elements [113,114,115,116].

Molecular tools must be selected based on the specific dimension of inoculant fate being investigated [3,6]. Persistence and abundance require strain-specific quantitative assays based on unique markers. Although ddPCR enables absolute quantification at low copy numbers, it requires inhibition controls and cautious interpretation of near-limit signals [93,94,95,96]. Dispersal assessments necessitate spatially resolved sampling designs, as single cores rarely capture colonization in heterogeneous soils [12,91]. For viability assays to be informative, matrix-specific validation and full reporting of parameters are required. Determining functional retention requires a shift toward activity-based measurements and pathway confirmation, including expression of critical steps, detection of transformation products, and isotopic approaches if attribution is needed [50,62,90,102,117]. Tracking the fate of inoculants cannot substitute for evaluating bioremediation performance, and its role is limited to clarifying mechanisms and constraints [7,18]. Evidence of inoculant disappearance alongside persistent functional signals and niche restructuring [83,84,107], as well as cases of extensive community remodeling without improved degradation [46,79,108], indicate the same conclusion. Inoculant fate can be interpreted only when it is associated with evidence of activity and when progression is chemically confirmed. Otherwise, it remains a description of presence without agency [1,6].

Ultimately, field relevance depends on explicitly defining what constitutes sufficient evidence for each dimension of inoculant fate. Persistence and abundance signals near analytical limits remain inconclusive in the absence of inhibition controls. Dispersal claims must be spatially replicated, and matrix-specific validations are needed to support viability evidence. Functional retention is the most stringent criterion and cannot be inferred solely from DNA-based detection. SIP directly links contaminant-derived atoms to active populations, thereby providing decisive evidence when functional agency is uncertain or contested. Therefore, each dimension of inoculant fate requires its own evidentiary threshold, and conflating them leads to systematic overinterpretation of inoculant performance in soils. For practical field applications, the key monitoring recommendation is to avoid collapsing inoculant fate into a single abundance metric. At minimum, field designs should distinguish persistence from functional retention and should be spatially replicated enough to capture patchy establishment. Recent work on recurrent inoculation underscores that survival can improve only under specific community and disturbance contexts [118], whereas chemically challenging fluorinated substrates show that proof of function may require product-resolved confirmation such as fluoride release rather than simple detectability [119]. Inoculant monitoring becomes mechanistically informative only when abundance, viability, and verified pathway output are interpreted together.

## 5. Functional Gene Abundance as an Indicator of Process Potential

In soil bioremediation, increases in functional gene abundance are often interpreted as evidence of enhanced degradation. This practice is convenient during early assessments, but it fails when gene counts are used to estimate process rates or to identify active degraders [6,110]. Gene abundance reflects metabolic capacity encoded in the metagenome, not actual flux. It shows whether a pathway exists and whether selective pressure maintains it, but it cannot verify pathway activation or the completion of contaminant turnover [3,62,64,120].

Marker choice is central to obtaining interpretable signals. Common markers such as *alkB* can be useful, yet their diagnostic value diminishes in chronically contaminated soils where gene signals may diverge from the bioavailable fraction [80]. Alternative enzymes, including CYP153, initiate the same reaction, making single-gene markers insufficient [30]. For PAHs, ring-hydroxylating dioxygenase markers function similarly [47], but broader coverage with markers such as *pahE* improves detection across environments [48,49]. Narrow panels risk missing key degraders, while overly broad panels inflate apparent potential when bioavailability constrains reaction rates. Systems with well-resolved and modular pathways, such as atrazine degradation, illustrate how marker specificity (for example, *atzA* encoding atrazine chlorohydrolase) enhances interpretive precision, yet gene presence alone cannot predict degradation rates because induction and substrate availability control pathway activation [61,62,120]. Interpretation becomes even more complex in bioaugmentation. Increasing gene counts may signal horizontal transfer rather than activity of the introduced strain. Strain-specific markers or independent evidence of pathway progression are required to verify functional retention [107]. In linuron degradation, for example, *PromA* plasmids transfer catabolic genes across diverse hosts, so increases in gene abundance may represent diffusion of function rather than the growth of a specific degrader [113]. When mobility and resistance determinants are co-selected, functional gene signals must be interpreted within the broader dynamics of horizontal gene transfer and selective pressure [114,115,116].

Measurement practices strongly influence whether copy numbers carry biological meaning. qPCR remains standard, while ddPCR offers absolute quantification and improved performance at low copy numbers [93]. However, soil-derived inhibitors and near-limit signals can result in poor precision in both approaches [95]. Reverse-transcription ddPCR shows higher tolerance to inhibitors and extends the range of quantifiable samples [96]. Regardless of platform, inhibition testing, recovery controls, and transparent normalization are essential. Normalization methods diverge widely across studies. Gene copies may be reported per gram of dry soil, per unit of DNA, or relative to 16S rRNA depending purpose, and each approach introduces distinct biases [2,113]. Relic DNA inflates DNA-based normalization [11,12,13,14], while variability in 16S normalization is the result of copy-number differences and community shifts [2,91,92]. False negatives remain an underrecognized limitation. Primer design is the primary source of failure, especially for diverse gene families. Degenerate primers may miss relevant taxa, while inhibitors can suppress amplification and mimic true absence. Primer coverage, database alignment, and inhibition testing should be clearly documented. Multimarker panels mitigate undercounting but require careful normalization to preserve biological meaning. Reporting per gram of dry soil often provides a more stable ecological reference. When 16S normalization is used, expected copy-number ranges or sensitivity analyses should accompany the data. Common quantification and normalization choices, along with their interpretive implications and major sources of bias, are summarized in Table 1. 

Although absolute transcript abundance per gram of dry soil remains the most conservative primary reporting metric, alternative normalization strategies can be informative when used explicitly as secondary analyses. Reporting per unit RNA, relative to 16S rRNA transcripts, or per cell equivalent may help address specific questions about transcriptional enrichment, growth dilution, or biomass-normalized activity. However, each denominator introduces distinct assumptions and failure modes. For that reason, these alternative normalizations should be interpreted as sensitivity analyses rather than as replacements for absolute reporting. From a monitoring perspective, functional gene abundance is most useful as a screening layer and not as a direct proxy for flux. The practical concerns relate to whether the selected markers capture the likely executors of both pathway initiation and completion, whether false negatives have been constrained, and whether normalization preserves ecological meaning. When these conditions are not met, gene abundance should be interpreted as a weak descriptor of potential rather than as evidence of ongoing degradation.

## 6. Gene Expression as Evidence of In Situ Activity

Information on genetic potential alone is no longer sufficient; the key concern is whether degradative activity occurs under in situ soil conditions. In this context, the transition from DNA-based analyses to RNA-based measurements is a turning point in bioremediation monitoring [111,112]. Gene abundance merely indicates the presence of relevant catabolic modules; in contrast, expression data reveal whether degradative machinery has been activated in the environment [2,3]. Numerous studies have shown that tracking transcripts of pesticide-degrading genes can indicate biodegradation in soil because RNA levels respond dynamically to contaminant exposure and changing environmental conditions [61,62,64,120]. Nonetheless, transcription is not synonymous with mineralization. Although transcription provides evidence of enzymatic engagement, it does not demonstrate pathway completion, particularly when bioavailability, oxygen limitation, or electron acceptor availability constrain reaction rates [6,25].

Atrazine is a canonical example of how expression data refine pathway interpretation. Expression of *atz* genes in the soil varies with substrate availability and microhabitat conditions, providing mechanistic insights beyond DNA detection alone [61,64,121,122]. Expression is the decisive criterion that distinguishes capacity from activity, facilitating assessment of whether degradation occurs in situ rather than only in controlled laboratory settings [15,62,63]. This increased resolution comes at the cost of heightened analytical sensitivity. Without stable normalization, disciplined sample handling, and rigorous quality control, comparisons among treatments become fragile and prone to artifacts [6,91].

In practice, reverse-transcription quantitative PCR (RT-qPCR) is predominantly used for transcript quantification and is increasingly complemented by reverse-transcription ddPCR. Owing to the pervasive presence of PCR inhibitors in the soil, successful amplification alone does not guarantee quantitative reliability [95]. Reverse-transcription ddPCR is highly tolerant to inhibitors derived from soils and plant material, which is advantageous when transcript levels approach the detection limit [93,96]. Nonetheless, investigations on the application of ddPCR in environmental matrices have consistently reported that variability often originates from sample preparation and extraction rather than from the amplification reaction itself [93,94,95,123]. Therefore, the reliability of expression-based inference depends on the entire analytical chain, including sampling, stabilization, extraction efficiency, inhibition testing, recovery controls, and transparent reporting [6]. Sampling designs pose difficulties for transcript-based analyses. RNA responds rapidly to short-term environmental fluctuations, and soils are inherently heterogeneous. When sampling schemes that explicitly address spatial and temporal variability are unavailable, transcript measurements may reflect microhabitat heterogeneity rather than genuine alterations in pathway engagement [2]. Best practices developed in microbiome research, therefore, apply directly, requiring adequate biological and spatial replication and explicit reporting of analytical procedures [1,91]. Although such approaches are not yet routine monitoring tools, systems–biology strategies may expand the range of experimentally testable degradation pathways for highly persistent contaminants. CRISPR (clustered regularly interspaced short palindromic repeats)-based functional screening, rational consortium design, and model-guided pathway engineering can help identify candidate modules that should later be evaluated under soil conditions with appropriate chemical and isotopic verification [124].

Furthermore, interpretation relies on what is accepted as evidence of activity. Detection of transcripts corresponding to initial pathway steps may seem compelling, yet such signals are often misleading in soils, as pathway initiation does not ensure downstream completion. Bottlenecks may arise at later stages or from local redox constraints [3,62]. Hence, tracking sets of transcripts spanning critical pathway steps, guided by hypotheses about likely rate-limiting reactions, is a more robust strategy [62,120]. Regarding aromatic compounds, PAH degradation is modular, and downstream reactions are important for interpreting activity [3,50,79,110].

Bioaugmentation introduces an additional interpretive challenge, i.e., attribution. Both the inoculant and native microorganisms that harbor homologous genes may produce identical expression signals, and shifts in community composition may redistribute degradative functions [83,84,109,117]. In such contexts, RT-qPCR alone is rarely adequate. Strain-specific markers, metatranscriptomic approaches that enable taxonomic assignment of transcripts, or isotopic techniques that can identify populations capable of assimilating the substrate are necessary [52,56,90,102]. Nonspecific induction further complicates interpretation because structurally related compounds, stress responses, or cosubstrates may upregulate degradative genes. Environmental manipulations that alleviate metabolic constraints may thus enhance biodegradation without direct induction by the target contaminant [25,89]. Transcript data must consequently be interpreted in conjunction with chemical measurements and environmental context [6].

A minimal expression-based demonstration of activity requires reproducible, quantitative detection of transcripts for pathway-critical steps under exposure conditions. This detection must be supported by inhibition testing, recovery controls, and biologically meaningful normalization [62,93]. In soils, selecting a stable reference target is challenging because community composition often shifts during treatment. Therefore, conservative practice favors reporting absolute transcript abundances, such as per gram of dry soil, and complementing them with parallel biomass measurements to distinguish activity from abundance effects [2,91]. Expression data do not constitute evidence of pathway completion or confirm that contaminant loss results from biodegradation. Transformation products and mass balances must be measured chemically, and where attribution is essential, isotopic approaches must be used to obtain decisive evidence. SIP links contaminant-derived carbon to active populations, distinguishing them from the full pool of genes and transcripts [53,90,99,125]. Protein SIP advances this logic by tracking the incorporation of isotopes into active enzymes [102,125]. Therefore, although expression provides a strong biological signal, its full scientific value can be derived only when it is paired with pathway-level evidence and chemical confirmation of the transformation [1,6]. Bulk soil enzyme activities also deserve a defined place in the framework. Dehydrogenase, urease, phosphatase, arylsulfatase, and related assays can reveal whether contaminant exposure or remediation treatment alters broader microbial metabolism in the C, N, S, and P cycles. These measurements can therefore serve as contextual indicators of in situ biological activation. They are not, however, pathway-specific and should not be interpreted as proof that the target contaminant is being transformed unless they align with molecular and chemical evidence. Analytical strategy determines whether multi-omics integration remains explanatory or becomes merely cumulative. In practice, robust designs combine curated pathway hypotheses with co-assembly or genome-resolved assembly, quality-screened metagenome-assembled genomes, transcript mapping to pathway-critical modules, and targeted metabolomics or transformation-product analysis aligned with predicted intermediates. Where feasible, proteomic evidence should focus on enzymes that define branch points or likely bottlenecks rather than on broad protein inventories. This staged integration reduces the risk that annotation completeness will be mistaken for pathway operation.

Both analytical choices and intrinsic system variability, therefore, influence the interpretation of expression data in soils. Because transcript signals are highly labile and spatially structured, observed differences may reflect microhabitat heterogeneity rather than true changes in pathway engagement. Sampling designs should explicitly define spatial units, replication levels, and timing relative to exposure or amendments. In addition, they should establish that observed expression differences exceed background variability. Most importantly, expression must not be conflated with reaction flux. Transcription indicates pathway engagement, not throughput. When transcripts increase without a corresponding accumulation of expected transformation products, interpretation should shift toward downstream bottlenecks, bioavailability constraints, or nonbiological processes that affect contaminant concentrations. Recognizing this distinction is essential to avoid overinterpreting expression signals as evidence of effective biodegradation. At this stage, expression alone is insufficient to establish pathway operation, necessitating explicit pathway reconstruction and verification.

## 7. Pathway Reconstruction and Multi-Omics Confirmation

**Figure 3:**
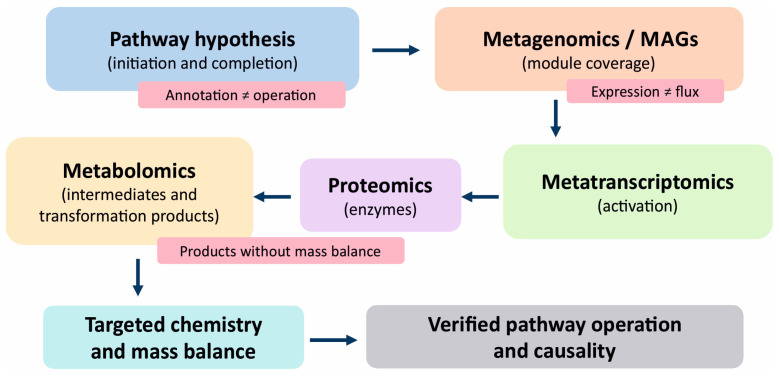
Multi-omics workflow for pathway verification. Pathway confirmation proceeds from a testable pathway hypothesis to molecular evidence of pathway occupancy and then to transformation-product and mass-balance evidence consistent with the predicted route. MAGs: metagenome-assembled genomes.

If monitoring is to connect inoculant fate, gene expression, and contaminant transformation in a defensible way, establishing that genes are present or that transcripts are detectable is not sufficient. It is necessary to reconstruct a credible pathway operating in the studied soil, identify the populations that carry its modules, and demonstrate that the pathway is consistent with the transformation products expected from it. According to this logic, omics approaches serve as evidentiary tools only when anchored in environmental chemistry and tested against intermediate steps, rather than treated as self-sufficient readouts [2,3]. Metagenomics is most informative when it moves beyond inventories to map reaction sequences, identify critical steps, and assign pathway modules to the populations most likely to exhibit reaction flux [126,127,128]. Investigations have underscored that reconstructing pathways and assigning roles within consortia improve both process design and interpretation, particularly for xenobiotics whose degradation is distributed across community members [110,128,129,130]. Nevertheless, annotation is not confirmation. Identical genes can function in distinct physiological contexts, and in soils, the outcome is often shaped by bioavailability and microscale redox heterogeneity that decouple sequence-based potential from pathway throughput [127,128,131,132,133]. The stepwise verification logic linking pathway hypotheses, multi-omics layers, and targeted chemical confirmation is illustrated in Figure 3. 

Successful multi-omics integration depends on analytical discipline at each stage of the workflow. DNA, RNA, protein, metabolite, and chemical measurements should be measured from temporally matched samples or from rigorously justified paired samples. Integration should be pathway-centered rather than taxonomy-centered, linking genes to transcripts, proteins, intermediates, and final products through explicit gene–protein–reaction logic. Assembly, binning, annotation, database choice, feature filtering, and metabolite identification criteria should be reported transparently because apparent biological differences may otherwise reflect pipeline decisions rather than pathway behavior. In practice, the strongest inferences arise when omics layers converge on the same pathway hypothesis and the chemistry confirms that the predicted transformations occurred.

In practice, pathway reconstruction operates at two linked levels: the functional annotation of read pools and assembled contigs to establish plausible module coverage and the use of metagenome-assembled genomes (MAGs) to attribute pathway modules to specific genomes and to distinguish pathways completed within a single organism from those partitioned across community members, which is common for many xenobiotics [1,3]. In pesticide systems, gene identification is therefore only the starting point for determining which organisms carry the relevant modules and under which conditions they execute the reactions, as evidenced by the cases of atrazine and phenylurea herbicides [65,121].

Metatranscriptomics adds the critical dimensions of time and context by showing which modules are activated in specific microniches and which remain latent potential [111,112]. In phenanthrene degradation, transcriptional responses can span a broad community and ecosystem rather than being dominated by a single executor [58]. In rhizosphere systems, metatranscriptomics has been used in multi-organism frameworks to capture how plants, fungi, and bacteria jointly shape functional responses to contamination, informing concepts of rhizodegradation and phytostimulation [112,134,135,136,137]. Even so, a metagenome paired with a transcriptome does not confirm pathway activity in the absence of chemical evidence or when it is inconsistent [138].

This limitation encourages multi-omics designs that complement genomics and transcriptomics with proteomics and metabolomics. For PAHs, when genetic potential aligns with protein and metabolite evidence, it helps localize bottlenecks, detect intermediate accumulation, and distinguish genuine detoxification from apparent shifts in the chemical form [3,79]. Metabolomics is particularly significant because transformation products must be identified and quantified to avoid misattributing contaminant loss to nondegradative processes. This approach is especially essential when sorption is strong or when rapid conversion produces forms that evade routine monitoring or persist in the matrix [98,133,138,139,140,141]. Proteomics strengthens the inference by identifying which enzymes are produced, which is advantageous in soils where transcript stability is limited and translation is strongly modulated by stress [3,102]. Protein SIP extends attribution by linking isotope incorporation to labeled proteins and the metabolic pathways that assimilate contaminant-derived atoms [90,102]. In sulfamethoxazole studies, combining DNA SIP with protein SIP has helped identify dominant degraders in soil, confirming the value of this strategy when DNA signals alone are ambiguous [72,125,142]. Parallel tracking of sulfonamide responses at the transcript and protein levels is also beneficial for observing resistance and degradation functions, especially when degradation intersects with selective pressures [73,143,144,145].

The limitations of pathway reconstruction are primarily attributed to data properties and the soil matrix itself [146,147]. Annotation quality depends on database coverage, and many xenobiotic-degrading enzymes remain underrepresented. This situation necessitates the development of predictive tools that infer candidate bioremediation enzymes from high-throughput data. Such tools are predictive, and their output is not a substitute for evidence of a process [110,148]. Moreover, omics outputs are sensitive to analytical decisions. Microbiome best practices explain how normalization choices and inference artifacts arise when the study design fails to account for spatial and technical variability [91,92]. In DNA-based omics, relic DNA can preserve historical signals that confound the interpretation of temporal change as a functional difference [12,146,147,149,150]. Genome-scale metabolic models can complement pathway reconstruction by testing whether annotated gene sets are stoichiometrically capable of supporting the proposed transformation under site-relevant nutrient and electron-acceptor conditions. In this role, models are hypothesis-generating rather than confirmatory. They can identify candidate bottlenecks, highlight missing reactions, and guide targeted metabolite measurements, but pathway verification still requires chemical and, where necessary, isotopic evidence [151].

Hence, the most productive strategy is not to add more data layers but to formulate a testable pathway hypothesis and verify it. Metagenomics should establish module coverage and organization within MAGs, metatranscriptomics should confirm activation of these modules under exposure, and metabolomics should document intermediates and products consistent with the predicted trajectory [3,81,110,130]. When the function is redundant and distributed, isotopic strategies bridge the attribution gap by shifting proof from sequence similarity to the incorporation of contaminant-derived atoms into biomass and functional molecules [1,90,102]. This evidentiary discipline prevents naive inference from DNA alone and the assumption that additional omics layers automatically increase certainty [2,91]. Evidentiary coherence is the decisive criterion in bioremediation, and for omics to be persuasive, it should be integrated with chemistry and interpreted with a strict separation of potential, activity, and verified pathway operation [6,18,79].

Comparability across studies depends on transparent bioinformatics processing and pathway annotation. The processing, assembly, and annotation steps, including software versions, key parameters, databases, and gene-to-pathway mapping rules, should be explicitly documented. Only then will apparent differences in pathway structure reflect pipeline choices rather than biology. When MAGs are used to attribute pathway modules to populations, claims of functional capacity should be accompanied by completeness and contamination metrics. Pathway reconstruction alone does not confirm a pathway. When metagenomic data suggest complete pathway coverage but metabolomic evidence remains inconclusive, the burden of proof shifts to operational verification. In this situation, efforts should be made to demonstrate that the predicted enzymes are detected at the protein level and that the expected intermediates or products are present at concentrations consistent with the proposed reaction flux. This evidentiary logic can be illustrated conceptually. In chronically contaminated soils amended with phenanthrene, metagenomic and marker data may indicate potential, and transcript analyses may signify activity during early exposure. Nonetheless, causal inference requires convergence with downstream intermediates and the isotope-based identification of active taxa. If intermediates accumulate without complete mineralization, interpretation should shift toward downstream bottlenecks rather than claims of complete bioremediation. This example illustrates how the framework gates inference at each tier and moves beyond reliance on simple before-and-after concentration changes. Across contaminant classes, the same evidentiary principle recurs: mechanistic resolution improves only when molecular observations converge with pathway-consistent chemistry. In phenanthrene systems, DNA or transcript data alone may suggest broad potential, whereas metabolite profiles and SIP resolve the active executors. In atrazine systems, pathway-specific genes and transcripts become far more informative when interpreted alongside hydrolytic intermediates and pathway completion logic. Recent fluorinated contaminant studies also reinforce why chemistry remains indispensable: defluorinating activity was accepted only when fluoride release and product-level analysis supported the proposed route [119].

## 8. SIP for Identifying Active Degraders

**Figure 4:**
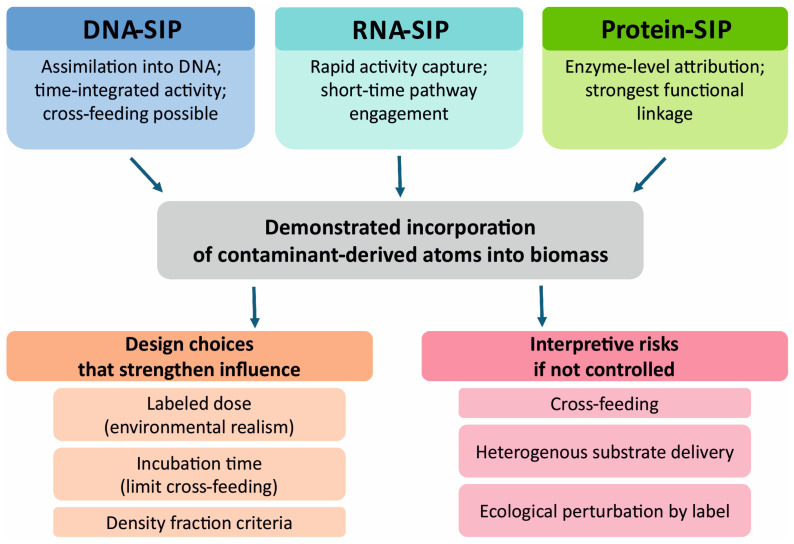
Stable isotope probing (SIP) modalities, design levers, and interpretive risks. SIP is most informative when the question is attribution, but its design must explicitly control for cross-feeding, dose perturbation, and ambiguous enrichment thresholds.

Attribution, i.e., determining which organisms actually use a contaminant as a source of carbon or energy in situ, is the most challenging aspect of soil bioremediation monitoring [1,3]. DNA- and expression-based approaches are easily confounded by functional redundancy, and in bioaugmentation, signals from the inoculant and native microorganisms often overlap [83,84]. SIP changes the logic of proof: rather than inferring activity from presence, it links activity to the physical incorporation of atoms from a labeled substrate into biomass, separating potential from process and strengthening causal inference [1,90,102]. The attribution logic of DNA, RNA, and protein SIP, along with key design choices and interpretive risks, is summarized in Figure 4.

In DNA SIP and RNA SIP, a substrate is most often labeled with ^13^C, nucleic acids are separated by density-gradient centrifugation, and the isotopically enriched fractions are analyzed [1,90]. RNA SIP responds more rapidly and better captures episodic activity, which is particularly relevant in heterogeneous soils [53,55,90]. Protein SIP moves the evidence to the level of translation by linking isotope incorporation to specific proteins, i.e., to the active enzymatic machinery. This shift is decisive when transcription does not translate linearly into enzymatic activity [102,125].

SIP is particularly valuable in ecologically ambiguous systems [1]. In the degradation of PAHs, similar catabolic modules are shared across many taxa, and SIP identifies truly active native degraders rather than merely marker-gene carriers [46,79]. SIP-based studies have helped identify uncultivable community members involved in pyrene biodegradation [90,125]. Such studies have also identified bacteria actively degrading phenanthrene that were not apparent from DNA profiles alone, highlighting habitat specificity among the executors of the process [52,53,152]. In biostimulation and rhizodegradation settings, SIP has provided information on communities involved in degrading polychlorinated biphenyls in soils subjected to plant and flooding treatments. In these systems, conventional signals confound plant-driven responses with contaminant-driven responses [125,137,153].

The value of SIP is particularly evident in bioaugmentation, where it enables separating the inoculant’s contribution from that of the native microbiome. This advantage also applies in cases where the inoculant acts indirectly and other populations ultimately assimilate contaminant-derived carbon [109]. DNA SIP has shown that trophic interactions and biotic pressure can reorganize the inoculant’s contribution to active degradation, reinforcing the idea that inoculant fate does not equate to functional agency [84,109]. For phenanthrene, RNA SIP has helped distinguish active degraders in free and biochar-immobilized forms, signifying that microenvironmental context can shift the executors, although functional potential remains similar [55,81]. In pharmaceuticals, SIP mitigates the risk of confusing biodegradation with sorption or partial biotransformation [98]. Combining DNA SIP and protein SIP has proven beneficial for identifying dominant sulfamethoxazole degraders in the soil, with the protein layer strengthening evidence of activity and helping distinguish degradation from generalized stress responses [102,125,142]. Approaches combining stable isotopes with single-cell imaging have precisely identified plastisphere colonizers that can assimilate sulfamethoxazole, which is relevant in systems where microplastics alter substrate availability and biofilm architecture [86,88,154].

SIP requires rigorous experimental design to avoid conclusions that appear strong but lack attributional support. The principal risk is cross-feeding, defined as the secondary incorporation of the label by organisms consuming metabolites or lysis products produced by primary degraders. This risk can be mitigated by carefully selecting incubation times, interpreting density fractions conservatively, and integrating with omics approaches to verify that heavy fractions contain pathway modules consistent with the expected biochemistry [3,79,90]. A second constraint arises from soil heterogeneity and uneven delivery of the labeled substrate to microniches, blurring isotopic signals and necessitating spatial replication and careful attention to extraction representativeness [52,91,99]. A third risk is ecological perturbation caused by the labeled dose, especially when contaminants are present at trace levels. Experimental designs that minimize artificial enrichment and interpretive caution to distinguish realistic in situ activity from potential effects revealed only under elevated exposure are required to mitigate this risk [6,72,90]. Within the evidentiary chain, SIP serves as a bridge between expression and pathway confirmation and is most potent when isotopically enriched fractions concurrently show gene modules and transcripts aligned with the predicted pathway, along with chemical consistency in measured transformation products. SIP therefore works best as the core of an integrated evidence package rather than as an isolated experiment [1,3,79,110,130]. In this configuration, contaminant disappearance and the presence of relevant genes can be documented. Moreover, populations that incorporate contaminant-derived carbon into their biomass and the functional context in which this flux occurs can be identified [1,56,90,102].

The evidentiary strength of SIP ultimately depends on experimental design and transparent reporting. SIP investigations should thus record the labeled dose and its justification, the incubation window, quality control for density fractionation, and the criteria used to define isotopic enrichment. As cross-feeding can blur attribution, this risk should be addressed both experimentally, by appropriately reducing the incubation time, and interpretively, by verifying that isotopically heavy fractions contain gene and transcript signatures consistent with the hypothesized pathway. Particular caution is required when contaminants occur at trace concentrations under field conditions. In such cases, label additions should preserve environmental realism, and interpretations should clearly distinguish activity observed under elevated experimental exposure from that inferred under ambient conditions. These precautions can ensure that SIP strengthens, rather than overstates, causal inference in heterogeneous soil systems. Machine learning tools that predict xenobiotic-degrading enzymes from molecular structure and metagenomic information may complement SIP at the stage of hypothesis generation. Such tools can prioritize candidate taxa, reactions, or substrate-enzyme matches before isotopic experiments are designed [155]. However, they do not resolve in situ attribution on their own and should therefore be treated as complementary predictive tools whose outputs still require chemical and isotopic validation. Large-scale field application of SIP remains methodologically constrained. Major limitations include the cost and availability of labeled substrates, the large soil mass often required for density fractionation, the limited throughput of fractionation and downstream analysis, and the risk that label addition perturbs systems operating at environmentally realistic trace concentrations. Field heterogeneity introduces a further constraint because adequate spatial replication rapidly becomes expensive and logistically difficult. For these reasons, SIP is often most powerful at the field scale when used as a targeted confirmatory tool in nested hotspots, microplots, or mesocosms rather than as a routine whole-site monitoring layer.

## 9. Study Design, QA/QC, and Integration of Molecular and Chemical Evidence

Even the most carefully selected molecular markers lose interpretive value if the study design fails to account for the fact that soil is a mosaic of microniches in which microorganisms, contaminants, and redox conditions rarely co-occur within the same physical space. The quality of inference in soil bioremediation is therefore largely determined at the planning stage, specifically by sampling design, control selection, and the manner in which molecular evidence is integrated with chemical measurements [1,2,6]. At the field scale, the evidentiary design is constrained not only by biology but also by cost. Spatial replication, repeated temporal sampling, inhibition testing, transformation-product analysis, and isotopic confirmation all improve inference, yet each adds a substantial analytical and logistical burden. Because soil heterogeneity often exceeds treatment effects, underpowered field designs can create a false economy in which reduced sampling lowers cost but also makes interpretation unreliable. A defensible field program therefore requires explicit prioritization of the minimum evidence package needed for the claim being made. Best practices in microbiome research consistently demonstrate that even technically sound sequencing or qPCR analyses can yield artifact-driven conclusions in the absence of biological and spatial replication, appropriate process controls, and transparent reporting of analytical workflows [12,91,92]. In bioremediation, this risk is amplified because sorption and sequestration can mimic biodegradation. At the same time, detoxification depends on the formation and fate of transformation products rather than on the disappearance of the parent compound alone [97,98]. 

Sampling strategies must therefore reflect both spatial and temporal heterogeneity. A single sampling location cannot represent a field site, and spatial variance often exceeds treatment effects, particularly in bioaugmentation, rhizodegradation, or systems where amendments act locally. A minimal, defensible design includes spatial replication and a consistent temporal framework. For studies addressing dispersal and establishment, transects and vertical profiling are often informative [2,8,12,83]. Activity measurements impose additional constraints because RNA is labile. Hence, sample stabilization and rapid immobilization of the biological material are core QA/QC requirements rather than logistical details. Temporal sampling schemes should capture early adaptation and induction phases, as well as later stages that reveal bioavailability limitations and aging effects, since simple before-and-after comparisons do not distinguish underlying mechanisms. Evidence from hydrocarbon-contaminated soils indicates that both the rate and extent of mineralization differ substantially among soils, suggesting that denser sampling is warranted during the early phases of treatment [1,8,27,29,112].

The central interpretive challenge associated with DNA-based measurements relates to system memory. Relic DNA is often abundant in soils, inflates diversity estimates, and masks temporal change; hence, eliminating or accounting for this fraction can reveal dynamics that remain invisible in standard extractions. As many inferences depend on temporal contrasts, QA/QC plans should explicitly assess the sensitivity of conclusions to the relic DNA pool [11,12,13]. vPCR can minimize the amplification of DNA from cells with compromised membranes, but its strong reliance on matrix properties and protocol parameters necessitates careful validation and comprehensive reporting [103,104,105].

PCR inhibitors are a pervasive source of uncertainty in soils. QA/QC for qPCR and ddPCR must therefore include inhibition testing, recovery controls, and extraction efficiency assessments. Although ddPCR enables absolute quantification and tends to be less sensitive to inhibitors, it does not resolve interpretive challenges at the detection limit or in highly variable matrices, making inhibition controls indispensable [93,94,95]. For expression analyses, reverse-transcription ddPCR is highly tolerant to soil- and plant-derived inhibitors, broadening the range of samples suitable for reliable quantification [96,123]. In amplicon sequencing, inference quality depends critically on process controls and transparent filtering. High-resolution sequence-variant inferencing reduces some artifacts associated with classical operational taxonomic unit clustering. However, this approach cannot replace replication, negative controls, or extraction blanks, which are essential in high-biomass soils and in the case of a genuine contamination risk. Good practice, therefore, involves the systematic use of blanks and negative controls, along with explicit reporting of quality thresholds [12,91,92].

**Figure 5:**
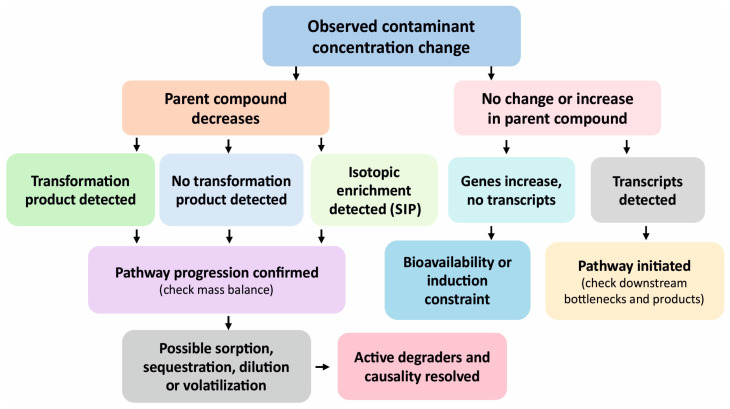
Decision framework for integrating molecular and chemical signals. The framework separates pathway representation, pathway engagement, and causal pathway operation so that apparent parent compound loss is not mistaken for verified biodegradation. SIP: stable isotope probing.

The most persistent challenges arise during the integration of molecular and chemical data. A practical linked-monitoring workflow begins with a site-specific question and an explicit pathway hypothesis. The next step is to select the minimum molecular and chemical layers required for the intended claim instead of maximizing data generation indiscriminately. DNA-based measurements are used to test pathway representation, RNA or protein data are used to test in situ engagement, and chemical analysis is used to test pathway progression and mass-balance coherence. SIP is introduced when attribution is uncertain, particularly in field systems with functional redundancy or inoculant-native overlap. The final interpretive step is to assign the result to the highest evidentiary tier justified by the data rather than to the most ambitious claim put forth by the investigator. A rule-based decision logic for reconciling molecular signals with contaminant fate and transformation products is provided in Figure 5. 

A decline in parent compound concentration alone is not evidence of biodegradation and may reflect sorption, sequestration, volatilization, dilution, or conversion to products outside the scope of routine monitoring. [1,3,6]. In soil–pharmaceutical systems, sorption and biodegradation often occur simultaneously, necessitating explicit separation of these processes and quantitative tracking of transformation products. For instance, degradation products of beta-blockers and carbamazepine can persist and retain environmental relevance, making product analytics integral to detoxification assessment. For sulfamethoxazole, rapid disappearance can coincide with slower, incomplete mineralization, demonstrating that parent loss is not a reliable surrogate for pathway completion [6,72,142,156].

Data integration should therefore follow explicit inference rules that preserve the distinction among potential, activity, and verified pathway operation. In the absence of a concentration decline, increased gene abundance points toward bioavailability constraints or a lack of induction rather than degradation. When transcripts are detected alongside the accumulation of transformation products, it indicates partial pathway activation with downstream bottlenecks rather than complete bioremediation. If the concentration declines without coherent product or activity signals, apparent processes and mass balance inconsistencies must be scrutinized. Where attribution is necessary, isotopic methods are helpful as they directly link contaminant-derived carbon to biomass and identify the populations responsible. This approach closes the inference gap that correlations between genes and concentrations cannot bridge [25,62,97,112,154]. Statistical design and normalization further enhance interpretive robustness. Reporting absolute quantities per gram of dry soil, alongside relevant physical parameters, is a conservative and transparent approach for molecular data. Sequencing workflows should avoid conflating relative and absolute changes, especially when biomass shifts occur. Time-series analyses should account for autocorrelation and site effects, and correlations should not be treated as substitutes for causal inference. Transparent reporting of statistical decisions and control of multiple testing are therefore paramount [2,12,91].

Associations between molecular markers and contaminant trajectories should be treated as descriptive unless competing explanations have been sufficiently disproven. Genes without activity do not establish process, transcripts without pathway-consistent products do not establish throughput, and parent-compound loss without coherent chemistry does not establish biodegradation. Stronger inference begins only when multiple lines of evidence reduce the set of plausible alternatives. Molecular monitoring can also inform quantitative process modeling when the measured variables are tied to explicit flux hypotheses. Gene and transcript data can be used to parameterize which pathway modules are present, inducible, or likely limiting, while chemical time series, electron-acceptor dynamics, and sorption descriptors constrain feasible reaction rates. In this setting, molecular data do not replace kinetic models; they improve model structure and help distinguish biological bottlenecks from mass-transfer limitation or abiotic loss. It is useful to separate descriptive association from stronger evidence. Gene detection supports a statement about pathway representation. Transcripts or relevant proteins support a statement about pathway engagement. Pathway-consistent transformation products, mass balance, and isotopic incorporation support stronger causal claims. Treating these evidentiary levels as interchangeable is one of the main reasons the results of molecular monitoring are overstated.

QA/QC requirements apply equally to analytical chemistry. When transformation products are part of the evidentiary chain, they should be identified and quantified in a methodologically sound manner, and their stability and subsequent transformations should be assessed. Otherwise, newly detected peaks may be overinterpreted as proof of degradation. Transformation products can persist and behave differently from their parent compounds, with direct implications for environmental risk assessment [6,97,98,156]. In practice, molecular and chemical monitoring should form a single, coherent evidentiary framework. A minimal, defensible linkage of inoculant fate, activity, and pathway operation requires meeting the following criteria: molecular signals must meet QA/QC standards for inhibition, controls, and biologically meaningful normalization; chemical analysis should encompass parent compounds and, where necessary, transformation products; and integration should preserve the analytical separation of potential, activity, and causality, strengthening attribution, with isotopic methods applied where necessary [3,90,91,95,97]. 

Ultimately, the quality of inference in soil bioremediation monitoring depends on study design, replication, and the transparent integration of molecular and chemical evidence. In heterogeneous field settings, a defensible design includes spatial replication across the treatment area and temporal coverage that captures both induction and plateau phases. Sampling schemes must record the spatial scale represented by each core and the distance between replicate cores. This documentation is necessary to interpret the observed variance ecologically rather than dismissing it as analytical noise. Furthermore, integration can benefit from predefined decision rules that specify acceptance criteria at each evidentiary tier. Genes detected without transcripts do not constitute evidence of activity. In the absence of transformation products, transcripts do not establish pathway operation. Similarly, product formation without coherent molecular support remains ambiguous unless mass balance closes. Defining these criteria a priori improves reproducibility and limits post hoc interpretation. Pilot testing under site-specific matrix conditions, including inhibitor profiling and assessment of detection limits for both molecular and chemical analyses, can strengthen the translation from laboratory methods to field application. Such pilot studies help calibrate sampling density, optimize extraction protocols, and ensure that analytical sensitivity matches expected contaminant concentrations. Statistical reporting must be transparent and include disclosure of the replication structure, units of analysis, variance components, criteria for outlier handling, and approaches to multiple testing control. In addition, absolute quantities per gram of dry soil should be reported to avoid scale ambiguity. Minimum QA/QC requirements and reporting expectations for the linked molecular monitoring workflow are shown in Table 2.

For practical application, five quantitative rules are especially important: (1) report absolute gene and transcript abundance per gram of dry soil as the primary quantitative metric; (2) use nucleic-acid-normalized, 16S-relative, or cell-equivalent metrics only as secondary sensitivity analyses, and state assumptions explicitly; (3) treat weak or near-threshold signals as inconclusive unless inhibition testing, recovery controls, and replicate concordance are satisfactory; (4) match spatial and temporal sampling density to expected heterogeneity rather than to analytical convenience; and (5) keep descriptive association separate from causal inference—genes indicate potential, transcripts indicate engagement, and pathway-consistent products or isotopic attribution establish pathway operation. This centralized guidance is intended to make the framework more portable across fields and experimental designs. Artificial intelligence- or machine learning-assisted data integration may become useful at this stage for feature ranking, multimodal clustering, pathway prioritization, and anomaly detection in large multi-omics plus chemistry data sets. Even so, model outputs should be treated as decision support. They do not relax the requirement for pathway-consistent chemical evidence and remain only as reliable as the metadata, calibration range, and mechanistic assumptions on which they are built [124,155].

## 10. Conclusions and Future Research Directions

The evidence synthesized in this review demonstrates that soil bioremediation for organic pollutants cannot be evaluated using a single data type. Chemical analyses alone may conflate biodegradation with sorption, sequestration, or transformation into products that are difficult to detect. In contrast, biological data interpreted without a chemical context may equate genetic potential with a process. Hence, linked molecular monitoring should be treated as an evidentiary architecture in which inoculant fate, functional gene abundance, expression profiles, and pathway reconstruction form complementary layers, each addressing a distinct question and each to specific sources of uncertainty [1,2,3]. Within this framework, the primary value of omics and quantitative molecular tools lies in alleviating causal uncertainty, especially in soils where degradation occurs within spatially constrained microniches and is governed by bioavailability, redox gradients, and community ecology.

The soil matrix warrants caution when interpreting molecular signals. Relic DNA introduces ecological memory that can distort before-and-after comparisons and inflate apparent changes in gene abundance [11,12,13]. Approaches such as vPCR, which distinguish live from dead fractions, can support inference but must be validated carefully and cannot serve as a substitute for evidence of activity or pathway operation [9,10,103,105]. QA/QC must therefore explicitly address inactive molecular fractions and the inherent variability of soil matrices [91,92]. 

Although functional gene abundance is a key indicator of pathway capacity and coverage, it does not indicate activity. For hydrocarbons, the interpretive value of markers such as *alkB* depends strongly on the availability of degradable fractions [30,80]. For PAHs, robust inference requires panels that capture pathway diversity and downstream steps, including *pah*-*rhd* and *pahE* markers [47,48,49]. In atrazine systems, well-characterized pathways improve marker interpretability, but they must be integrated with expression data and chemical confirmation [61,62].

In bioaugmentation, the fate and functional contribution of the inoculant often diverge. Interactions with native microbiomes, priority effects, competition, and biotic pressure shape establishment and functioning. These factors can explain both community restructuring without improved degradation and the functional handoff to native microorganisms [83,84]. Studies in hydrocarbon-contaminated soils emphasize this asymmetry between persistence and function [107,108]. When drawing conclusions about the causal contribution of the inoculant, evidence should extend beyond detectability to include pathway-level activity and the attribution of reaction flux.

Pathway reconstruction increasingly depends on integrating omics approaches with environmental chemistry. Metagenomics and metatranscriptomics can recover degradative modules and assign them to populations in hydrocarbon-, pesticide-, and rhizosphere-associated systems [117,121,128,157]. Even so, a reconstructed pathway remains hypothetical until corroborated by intermediates and products that are consistent with the mass balance [81,97,98,138].

Isotopic methods are the most definitive tools for attribution. SIP assigns contaminant-derived atoms directly to active taxa and functions, mitigating the risk of spurious correlations. DNA SIP and RNA SIP studies have repeatedly shown that active degrader communities can differ markedly from those inferred solely from DNA profiles, especially across anthropogenic gradients or when microenvironments are altered [53,55,90,101]. Protein SIP and single-cell isotopic approaches extend attribution to enzyme-level resolution. Thus, active degraders can be identified even within complex biofilms and plastisphere structures [86,125,154].

Nonetheless, several research gaps remain. Monitoring degradation at trace concentrations that better represent environmental conditions is a critical challenge. High-dose experimental designs may overestimate in situ activity, as evidenced by sulfamethoxazole mineralization and comparable pesticide studies [6,72]. Multistressor systems in which degradation occurs alongside selective pressures on resistance, gene mobility, and functional reorganization are another critical frontier. Microplastics and other stressors modify biofilm architecture, substrate availability, and pathway performance, which means that degradative and adaptive stresses should be assessed integratively [74,75,85,86,116].

Recent research on agrochemical remediation, enzyme-centered biodegradation, recurrent inoculation, fluorinated contaminant transformation, consortium-based remediation of per- and polyfluoroalkyl substances, genome-scale metabolic modeling, and machine learning tools for enzyme prediction points in the same direction. Mechanistic understanding improves when sequence-based evidence is linked to pathway chemistry, ecological context, and explicit interpretive rules. This is the standard that should be normalized in the field [4,5,66,67,118,119,124,151,155]. In this context, several research priorities stand out more clearly. First, pathway inference needs to be tested under environmentally realistic, low-concentration exposure rather than high-dose laboratory conditions. Second, stronger methods are needed for systems in which biodegradation intersects with resistance selection, horizontal gene transfer, and multistressor effects such as microplastics. Third, field deployment will depend on workflows that remain informative under high spatial heterogeneity, limited budgets, and uneven contaminant accessibility. Fourth, pathway prediction, synthetic consortia design, genome editing, and model-guided intervention should be pursued as hypothesis-generating tools, but they must remain anchored in verified transformation chemistry and realistic soil constraints [4,5,66,67,118,119,124,151,155]. Finally, harmonized QA/QC and reporting standards are needed if linked molecular monitoring is to become comparable across studies and defensible in field decision-making [89,147,148]. Reference soils, negative controls, and inhibition testing are vital, especially in systems with high matrix variability. A defensible evidentiary package that links inoculant fate, expression, and pathway operation must include the following three elements: reliable chemical characterization of parent compounds and transformation products, gene panels spanning pathway-critical steps with explicit matrix controls, and confirmed in situ activity supported by expression data and inhibition controls [48,83,90,96,97,120]. Minimal evidence packages required for defensible inference across common study contexts are synthesized in Table 3.

Ultimately, the aim of molecular monitoring is to organize uncertainty into a coherent chain of evidence. Only within this discipline can researchers extend beyond documenting contaminant loss to explaining mechanisms, identifying executors, diagnosing pathway bottlenecks, and designing interventions that shift a system from potential to realized reaction flux. The key contribution of this review is to formalize a practical, testable framework that explicitly separates degradative potential, in situ activity, and causal pathway operation. It also anchors these distinctions within a defined study design and QA/QC requirements. This framework integrates multi-omics with chemical verification and, where necessary, isotopic attribution, thereby clarifying when molecular signals support defensible causal claims in soils rather than merely being descriptive. Progression between evidentiary levels is explicitly gated so that conclusions at each stage condition interpretation at the next. In this way, inference control is prioritized over the accumulation of molecular signals, thereby strengthening the scientific and practical value of molecular monitoring in soil bioremediation.

## Figures and Tables

**Table 1:** Recommended reporting bases for gene and transcript quantification in soil bioremediation studies.

Category	Reporting Basis	Main Interpretation	Recommended Use and Essential Caution
Absolute soil-mass basis	Gene copies per g dry soil (DNA)	Pathway potential per soil mass	Best for field comparisons; requires dry mass normalization and strict controls to avoid bias
	Transcript copies per g dry soil (RNA)	In situ pathway engagement	Best for time-series analyses; requires rapid stabilization and RNA-quality controls because RNA is highly labile
Extract-normalized basis	Gene copies per ng DNA	Potential per extracted DNA	Useful for method comparisons but should be paired with per g dry soil values to avoid bias
	Transcript copies per ng RNA	Relative transcription within the RNA pool	Useful in controlled microcosms; pair with per g soil RNA yield and quality metrics to avoid bias
Community-relative basis	Gene/16S rRNA gene ratio	Functional capacity relative to the total community	Useful for assessing enrichment, but must be paired with per g soil absolute counts to avoid ratio bias
	Transcript/16S rRNA transcript ratio	Expression relative to total rRNA output	Best as a sensitivity check; pair with absolute transcripts and RNA-quality metrics
Biomass-proxied basis	Gene copies per cell equivalent	Potential per estimated cell	Useful for separating biomass change from enrichment; requires an independent biomass proxy and per g dry soil values to reduce uncertainty
	Transcript copies per cell equivalent	Activity per estimated cell	Useful for distinguishing induction from growth; requires replication and standardized stabilization due to error-prone RNA and cell-equivalent estimates
Analytical or trend-only basis	Copies per extract or reaction	Analytical comparability only	Suitable for validation only; must be paired with per g dry soil values and recovery metrics
	Fold-change relative to baseline	Direction of change	Useful for temporal comparisons but should be paired with raw values to avoid baseline-related bias

**Table 2:** QA/QC and reporting checklist for molecular monitoring workflows.

Workflow Step	Key Controls	Recovery/Inhibition	Report at Minimum	Main Risk and Mitigation
**Pre-analytical stage**				
Sampling and stabilization	Field blanks; replicate cores	Not applicable	Spatial scale; time to stabilization; storage conditions	Spatial heterogeneity: use spatial replication and rapid stabilization
**Nucleic acid extraction**				
DNA extraction	Extraction blanks; spike-ins	Recovery surrogate; dilution test	Yield per g of soil; extraction protocol	Relic DNA inflation: compare with PMA-treated material or perform sensitivity analysis
RNA extraction	RT-minus; extraction blanks	Recovery control; inhibition test	RNA yield; stabilization method; RT efficiency	RNA degradation: stabilize immediately and verify integrity
**Targeted quantification**				
qPCR/ddPCR (DNA)	No-template controls; standards	Dilution series; spike recovery	LOD/LOQ; assay efficiency; inhibition outcome	False precision near LOD: report limits and use replicate runs
RT-qPCR/RT-ddPCR (RNA)	RT-minus; no-template controls	Inhibition test; RT recovery	Absolute units; RT protocol; LOD/LOQ	Expression–flux mismatch: interpret together with chemistry
**Community and functional profiling**				
Amplicon sequencing	Extraction blanks; mock community	Not applicable	Filtering thresholds; read depth; ASV method	Contamination bias: subtract blanks and retain replication
Metagenomics/MAGs	Negative controls; coverage checks	Not applicable	Assembly metrics; completeness and contamination	Annotation does not prove activity: requires products or SIP support
Proteomics	Process blanks; technical replicates	Recovery, when applicable	Protein identification criteria; coverage	Low-abundance enzymes may be missed: use targeted confirmation
**Transformation evidence**				
Metabolomics/TPs	Analytical blanks; standards	Recovery; matrix effects	TP identification criteria; quantification limits	Product misassignment: confirm with authentic standards
SIP fractionation	Unlabeled control; density marker	Fraction recovery; gradient QC	Enrichment threshold; fraction definition	Cross-feeding: shorten incubation and verify pathway consistency

ASV: amplicon sequence variant; ddPCR: droplet digital PCR; LOD: limit of detection; LOQ: limit of quantification; MAGs: metagenome-assembled genomes; PMA: propidium monoazide; QC: quality control; qPCR: quantitative PCR; RT: reverse transcription; SIP: stable isotope probing; TPs: transformation products.

**Table 3:** Minimal evidence packages across common study contexts.

Study context	Primary objective	Minimum molecular layer	Minimum chemical layer	When SIP is essential	Likely failure point
Microcosm (oxic)	Activity	Functional genes + transcripts	Parent + key TPs	Redundant degraders; attribution required	Overinterpreting transcripts as flux
Microcosm (anoxic)	Potential/activity	Functional genes; limited transcripts	Parent only; selected TPs	Overlapping pathways; unclear executors	Missing downstream confirmation
Mesocosm (oxic)	Activity → causality	Genes + transcripts; targeted omics	Parent + TPs; partial mass balance	Multiple active taxa; inoculant vs. native	Community shifts masking executors
Mesocosm (anoxic)	Potential	Functional genes	Parent only	Rare degraders; pathway uncertainty	Gene presence ≠ activity
Field study (oxic)	Causality	Genes + transcripts (where feasible)	Parent + TPs; mass balance indicators	Attribution contested or regulated	Sorption mimicking degradation
Field study (anoxic)	Potential	Functional genes	Parent only	Attribution critical for risk decisions	Inaccessible intermediates
Biostimulation	Activity	Community genes + transcripts	Parent + TPs	Functional redundancy	Misattributing stimulation effects
Bioaugmentation	Causality	Strain markers + activity evidence	Parent + TPs; mass balance	Inoculant contribution questioned	Persistence ≠ functional retention
High concentration	Activity	Genes + transcripts	Parent + TPs	Executor identity uncertain	Bottlenecks overlooked
Trace concentration	Potential	Sensitive gene markers	Parent only	Low signal-to-noise	Dose-driven artifacts

SIP: stable isotope probing; TPs: transformation products.

## Data Availability

No new data were created or analyzed in this study.

## Abbreviations

*alkB*	gene encoding alkane 1-monooxygenase
*alkH*	gene which encoded alcohol dehydrogenase
*atz*	genes involved in atrazine degradation
*atzA*	gene encoding atrazine chlorohydrolase
ASV	amplicon sequence variant
CRISPR	clustered regularly interspaced short palindromic repeats
ddPCR	droplet digital PCR
LOD	limit of detection
LOQ	limit of quantification
MAGs	metagenome-assembled genomes
*pahE*	gene encoding polycyclic aromatic hydrocarbon hydratase-aldolase
*pah*-*rhd*	genes which encode polycyclic aromatic hydrocarbon ring-hydroxylating dioxygenases
PAHs	polycyclic aromatic hydrocarbons
PMA	propidium monoazide
QA	quality assurance
QC	quality control
qPCR	quantitative PCR
RT	reverse transcription
RT-qPCR	reverse transcription–quantitative PCR
SIP	stable isotope probing
TPs	transformation products
*trzN*	gene encoding a triazine hydrolase
vPCR	viability PCR
